# Fractures and Separated Epiphyses

**Published:** 1911-09

**Authors:** 


**Fractures and Separated Epiphyses.** By ALBERT J. WALTON, F.R.C.S. Pp. vii, 288. London : Edward Arnold. 1910.

This is a useful book, clearly written and well illustrated with good skiagrams, which will be valuable to those who have to treat fractures either in hospital or private practice. We think the pictures of the various bones, with the lines of fracture. indicated on them, very helpful.

The two extremes of treatment—the method in which splints are not used at all, so warmly advocated by Lucas Championnière, and the method of fixing all fractures by operation—are fully discussed, but we are glad to see that neither is advocated by the author. He, however, very wisely lays stress on the need for early passive movement and massage; but he is not quite clear in expressing his meaning as to the use of splints, for on page 23 he says : " The method of fixing an injured limb, such as a leg, firmly in splints for three or four weeks is to be strongly condemned." What he obviously means is that during this time massage, and to a certain extent passive movement, should be employed, for he tells us so elsewhere ; but surely he does not mean that he would remove a fractured leg, even with a tendency to troublesome displacement, from the back-splint, in order to carry out these measures during the first few weeks, or even allow it to lie with so little support upon it that displacement of the fractured end might occur. What he does probably mean is that he would remove the side splints for this purpose, or, in other words, that he would not keep the limb surrounded by splints so that it could not be reached for massage and passive movement.

His reference to the fact that a slight displacement of the ends of the bones seen in a skiagram is no more than we may expect with the best treatment short of open operation, and is not necessarily followed by a bad functional result, is very important. When surgeons began to examine skiagrams of fractures they had "set" they were very disappointed with the result, and yet again and again, though they could not get perfect apposition, they found that the patient had a very useful limb.

We are glad to see that the author, though he describes the method of treatment which consists in the immediate application of a plaster case to the limb, does not recommend it. Such a case, as he says, cannot be a good fit if it is put on when the limb is much swollen, when that swelling subsides, and if it is put on before the swelling comes on then the risk to the nutrition of the limb is very real.

In writing of the various complications of fracture—a valuable portion of the book—he recommends suture of a ruptured main artery, both in subcutaneous injury and also in compound fracture. In the former it may be very good treatment; but looking at the fact (as he himself admits) that our attempt to render a compound fracture aseptic often fails, and some amount of suppuration occurs, the risk of failure of union of a sutured main artery would appear to us to be too great to make such a proceeding advisable. His account of injuries of nerves in fractures is very good : it is evidently taken from the work of Mr. Sherren, to whom reference is made. We notice that he has forgotten to include analgesia, as well as the loss of the sense of extremes of heat and cold, under the term "protopathic" sensation.

The directions for diagnosis and treatment are given with regard to each fracture. Surgeons differ so much in the exact method they employ for particular fractures that we cannot expect the author to give all of these. He tells us simply the method which he recommends, and generally the reasons why we should adopt it. More than this we can hardly expect in a work of quite moderate size, which may be regarded as a useful guide in practice. Those of us who have had to treat many fractures may in some varieties prefer other methods of which we have had experience, but the methods advised by the author seem very reasonable, and have probably been well tried in the practice of the London Hospital.

